# Supplementary material for: Aptamer Selection Based on Microscale Electrophoretic Filtration Using a Hydrogel-Plugged Capillary Device
Source: Molecules. 2022 Sep 8;27(18):5818. doi: 10.3390/molecules27185818 (PMC9505737; doi:10.3390/molecules27185818)
Supplement: Supplementary file 1 [file molecules-27-05818-s001.zip › molecules-1801846-supplementary.pdf]

molecules

Supporting Information

Table S1. Sequences of the primers and the library used.

	Sequences (5′ – 3′)
Forward primer for selection	CCTACGGGCGGCAGCAG
Reverse primer for selection	GACTACCCGGGTATCTAATCC
Forward primer for sequencing	TCGTCGGCAGCGTCAGATGTGTATAAGAGACAGCCTACGGGCGG-CAGCAG
Reverse primer for sequencing	GTCTCGTGGGCTCGGAGATGTGTATAAGAGACAGGAC-TACCCGGGTATCTAATCC
Random sequence (N = A, T, G, or C)	FITC-GACTACCCGGGTATCTAATCC-N_{40}-CTGCTGCCGCCCGTAGG

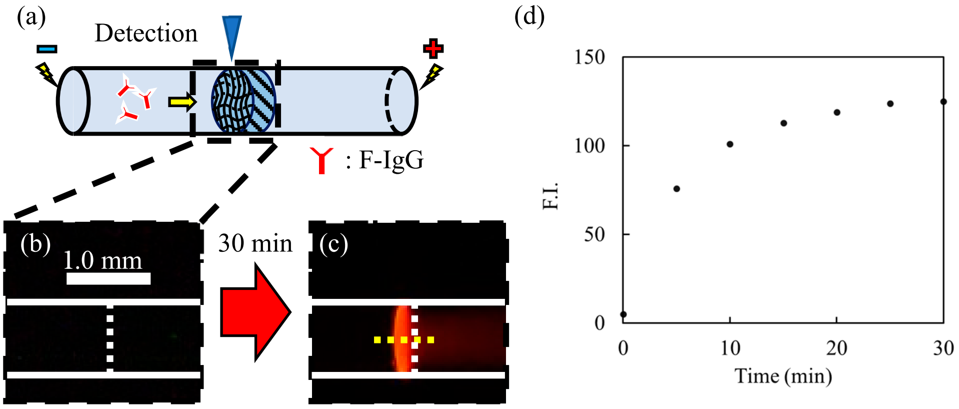

Figure S1. Confirmation of the entrapment of immunoglobulin G (IgG). (a) Schematic illustration and fluorescence images obtained near the upstream interface of the gel (b) before and (c) after the introduction of fluorescently labeled IgG. The vertical white broken lines represent the cathodic interface of the plugged hydrogel. Imaging conditions: exposure time, 2 s; filter, red fluorescent protein (RFP) for excitation, 540 ± 25 nm; RFP for emission, >572 nm; applied voltage, 100 V. (d) Relationship between the time of introducing the labeled IgG and fluorescence intensity, as estimated by the ImageJ software. The same ImageJ software was used to generate the estimates shown in (c) and (d).

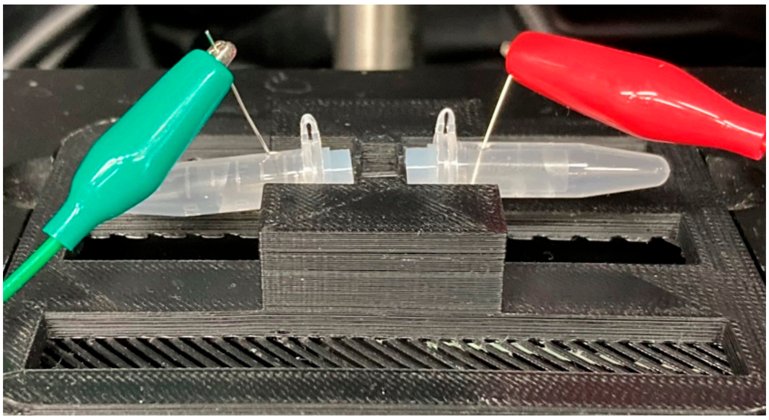

Figure S2. Photographs of the experimental setup. Both ends of the gel-plugged capillary were connected to the poly(dimethylsiloxane) (PDMS) reservoirs using a platinum electrode.

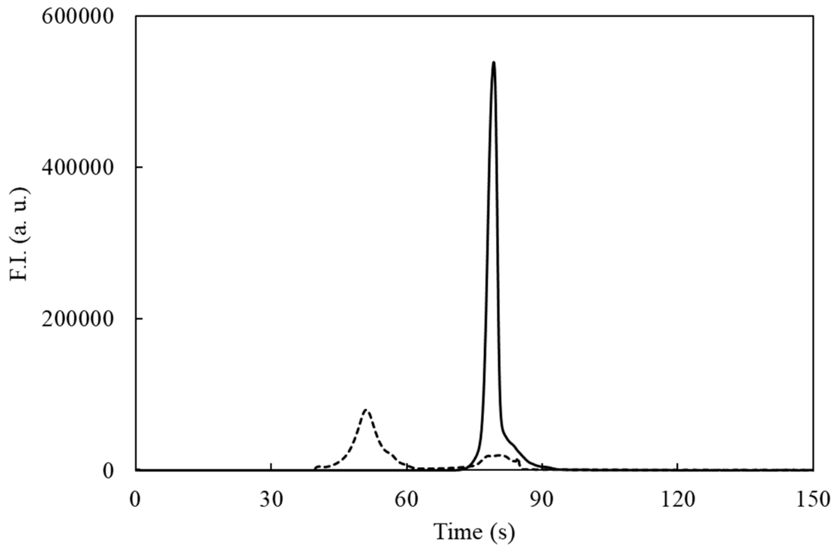

Figure S3. Analysis of the ability of the immunoglobulin E (IgE) aptamer to bind to IgE in 10 mM Tris-HCl buffer solution (pH 9.0) using CE-LIF. The lines represent 100 nM IgE aptamer (solid line), 10 nM IgE aptamer, and 80 nM IgE (broken line).

SI 1. Calculating LOD for S1on electropherogram results

First, the limit of detection (LOD) was calculated from the electropherogram to estimate the amount of S1 consumed by binding IgE (Figure 6b). After CE-LIF analyses of FITC-S1 at various concentrations, the fluorescence intensities were plotted against their concentration, and a calibration curve was estimated using a fitting software (Origin Pro). A linear fitted curve was obtained using the following equation:

$$\text{(fluorescence intensity)} = 2530 \times [\text{S1}] + 100. \quad \text{(S1)}$$

where the value of the intercept, 100, is the intensity of baseline noise. When the LOD value was defined as a signal-to-noise ratio (S/N) of three, the value was determined by substituting 300 (S/N = 3) on the left side of Equation S1. The LOD value of FITC-S1 was calculated to be 0.079 nM.

SI 2 *Evaluation of the binding ability of the aptamer candidates using nonequilibrium capillary electrophoresis of equilibrium mixtures (NECEEM) experiments*

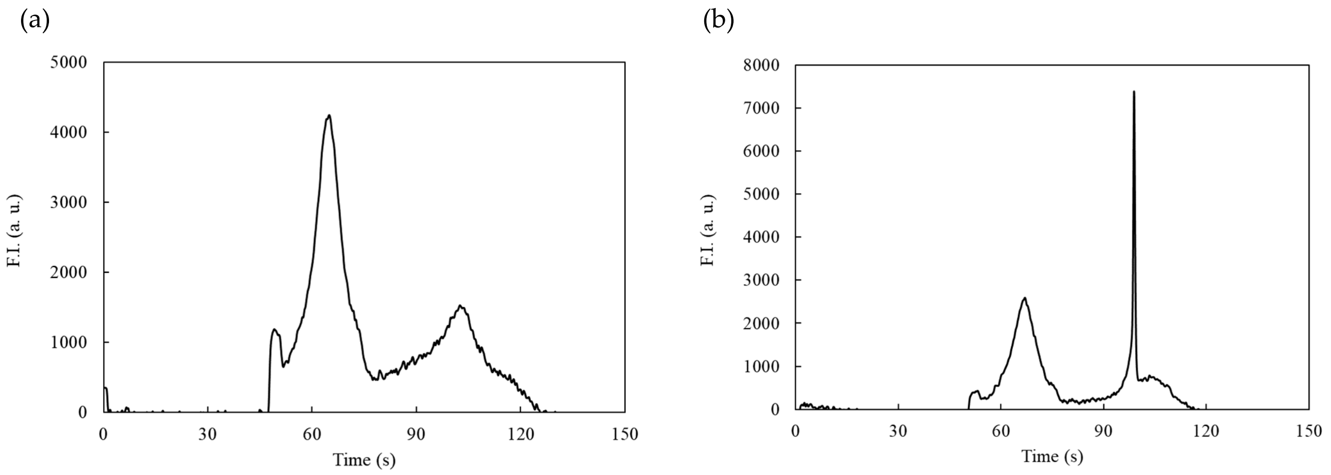

Figure S4. Analysis of the binding ability of S1 (a) or S2 (b) using CE-LIF under the NECEEM experimental conditions. The concentrations of FITC-S1 or -S2 and IgE were 25 nM each in 10 mM Tris-HCl buffer solution (pH 9.0). The other experimental conditions are the same as those shown in the main manuscript, except that the sample injection time was changed from 16 to 8 s.

To evaluate the binding ability (dissociation constant, K_D) of aptamer candidates, oligonucleotide S1 or S2 to the target molecule, IgE, the mixtures of FITC-S1 or FITC-S2 and IgE were analyzed on the basis of nonequilibrium capillary electrophoresis of equilibrium mixtures (NECEEM).

Solutions of FITC-S1 and FITC-S2, were prepared and mixed with IgE in 10 mM Tris-HCl buffer solution (pH 9.0). The final concentrations of FITC-S1, FITC-S2, and IgE were 25 nM each. The mixed solution was introduced into the capillary 4 h after mixing and NECEEM analyses were performed. Three peaks related to FITC-S1 were observed at 50, 65, and 105 s (Figure S4a), whereas another sharp peak appeared at 100 s in the case of the mixture containing FITC-S2 (Figure S4b). In Figure 6 in the main manuscript, it was confirmed that the peaks at 50 and 65 s represent the complex of IgE and FITC-S1 or FITC-S2. On the other hand, a broad peak related to free FITC-S1 or FITC-S2 was clearly observed at 105 s under these conditions, which were not present in the data on which Figure 6 was based. These results indicate that some oligonucleotides were not bound to IgE based on association/dissociation equilibrium due to the increasing and decreasing concentrations of S1 or S2 and IgE compared to those in Figure 6. The sharp peak observed at 100 s in Figure S4b also suggests that there are two types of oligonucleotide conformations: one is stable, such as hairpins and G-quadruplexes, and the other is unstable, such as linear. When oligonucleotide molecules are in conformational equilibrium, differences in their conformation affect their electrophoretic mobility, which broadened the width of the peak. Therefore, the sharp peak in Figure S4b suggested the existence of FITC-S2 with a stable conformation.

To discuss the above suggestion related to the stability of the conformation of S1 and S2, the variations in the Gibbs free energies (ΔG) by the formation of the most stable two-dimensional conformation were calculated by mfold. As a result, the values of ΔG were estimated to be -0.9 and -6.15 J mol^{-1} for S1 and S2, respectively. This indicates the more stable conformation of S2 than that of S1 under the experimental condition. Thus, the sharp peak in Figure S4b indicates that S2 was in a conformational equilibrium under these conditions. Generally, there are two types of aptamers:

one is an aptamer with a preorganized stable conformation for binding and the other is that it can bind the target by changing its conformation. Thus, whether the stable structure, unstable structure, or both are bound to IgE under these conditions should be considered.

To estimate the binding mechanism, K_D values were calculated from Figure S4 on the basis of NECEEM using the following equation S2:

$$K_D = \frac{B_{tot}\,(1 + A_{eq}\,/\,C_{eq}) - A_{tot}}{1 + C_{eq}\,/\,A_{eq}} \tag{S2}$$

where B_{tot} and A_{tot} are the initial concentrations of IgE and free FITC-S1 or FITC-S2, respectively, and A_{eq} and C_{eq} are the concentrations of the free oligonucleotides and complexes calculated from the heights of the peaks in the NECEEM electropherograms, as shown in Figure S4.

In the NECEEM analyses of the mixtures of 25 nM FITC-S1 or FITC-S2 and 25 nM IgE, the values of C_{eq} of FITC-S1 and FITC-S2 were estimated as the sum of the heights of the peaks at 50 s and 65 s. Related to the A_{eq} of FITC-S1, it was estimated from the peak heights at 105 s. On the other hand, the values of A_{eq} of FITC-S2 were estimated by each peak height or as the sum of the heights of these peaks at 100 s and 105 s based on the assumptions that the complexation depended only on the binding to either the stable or unstable conformation, or to both. As a result of the estimation using the above equation, the K_D values of FITC-S1 were calculated as 0.83 nM, whereas those of FITC-S2 related to either sharp or broad peaks, or to both were 28.4, 1.55, or 34.6 nM, respectively.

To assess the validity of the estimated K_D values, concentrations of FITC-S1 or FITC-S2 and IgE complexes were estimated under the conditions described in the main manuscript, with 10 nM FITC-S1 or FITC-S2 and 100 nM IgE, using the following equation:

$$K_D = \frac{[\text{FITC-S1}]\ [\text{IgE}]}{[\text{FITC-S1-IgE}]}\ or\ \frac{[\text{FITC-S2}]\ [\text{IgE}]}{[\text{FITC-S2-IgE}]} \tag{S3}$$

where [FITC-S1 (or -S2)], [IgE], and [FITC-S1 (or -S2)-IgE] are the concentrations of free FITC-S1 (or -S2), unbound IgE, and the complex under association/dissociation equilibrium, respectively.

As a result, [FITC-S1-IgE] was calculated as 9.91 nM. Thus, the calculated value of [FITC-S1] under equilibrium was 0.09 nM, which was almost consistent with the experimental results that the peak of FITC-S1 was not observed under these conditions (LOD, 0.079 nM). For FITC-S2, the values of [FITC-S2-IgE] were calculated as 7.65 or 9.83, or 7.28 nM using the previously described K_D values. Thus, it can be estimated that there were 2.35 or 0.17, or 2.72 nM FITC-S2 in each solution. Among these values of [FITC-S2], the data involving the sharp component, 2.35 and 2.72 nM, were much larger than the LOD (0.079 nM), which is inconsistent with Figure 6. On the other hand, the data estimated only from the broad component, 0.17 nM, was acceptable. These results suggest that the unstable component detected as the broad peak was primarily involved in binding, with little contribution from the stable component observed as a sharp peak. The reason why the sharp component was not observed in Figure 6 was the difference in time after mixing. In the case of the analyses described in the main manuscript, the measurements were performed a few days after mixing the solutions because the measurements were performed by a collaborator. Thus, it was assumed that there is sufficient time to reach the conformational and association/dissociation equilibria, resulting in the disappearance of the peak in Figure 6. On the other hand, the NECEEM analyses described above were conducted 4 h after mixing the solutions, which was probably insufficient to reach both the conformational and association/dissociation equilibriums of FITC-S2 and IgE completely because of the stable structure of FITC-S2 with a large ΔG value. Therefore, it was

concluded that the K_D values from the broad peaks of FITC-S1 and S2 in NECEEM were reasonable for evaluating their binding ability to IgE.

From the above discussion, the following binding scheme is proposed (Scheme S1). Before mixing, the stable and unstable conformations of S1 and S2 were in equilibrium in the solution under the experimental conditions. When S1 or S2 was mixed with IgE, S1 or S2 in an unstable conformation was stabilized by binding to IgE, which reduced their concentration without binding. As a result, the concentrations of S1 or S2 with a stable conformation also decreased owing to the variation in the conformational equilibrium. In the case of S1, the conformation could easily change because of the small value of ΔG, so that the sharp component rapidly disappeared as shown in Figure S4. In the case of S2 with the stable conformation, on the other hand, the stable conformation without binding remained 4 h after mixing owing to the large value of ΔG. Binding of S2 to IgE could also be completed with sufficient time for both conformational and association/dissociation equilibria, as shown in Figure 6 in the main manuscript. Further experiments are required to elucidate the mechanism in detail; however, this is an issue for future studies, as this is not the major subject for the development of the novel aptamer selection method.

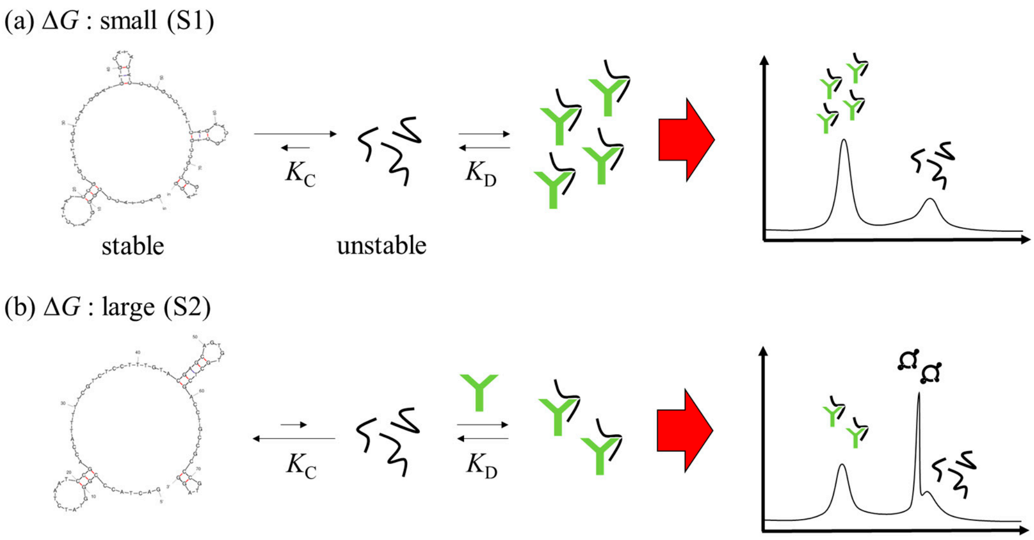

Scheme S1. Illustrations of possible binding schemes based on both conformational and association/dissociation equilibriums of S1 (a) or S2 (b). K_C is the conformational equilibrium constant. (a) When the value of ΔG is small, the conformational equilibrium will be negligibly small. (b) When the value of ΔG is large, the sharp peak from the free oligonucleotides could be observed due to their own stable conformation.

Reference:

[37] Victor, O.; Svetlana, M. K.; Sergey, N. K. Nonequilibrium Capillary Electrophoresis of Equilibrium Mixtures, Mathematical Model. *Anal. Chem.* **2004**, *76*, 1507–1512. https://doi.org/10.1021/ac035259p.
